# Skull Base Osteomyelitis: A 5‐Year Review and Prognostic Outcome in a Single Tertiary Institution

**DOI:** 10.1002/oto2.70001

**Published:** 2024-08-28

**Authors:** Liew Yew Toong, Sakina Ghauth, Ng Yin Xuan

**Affiliations:** ^1^ Department of Otolaryngology Universiti Malaya Kuala Lumpur Malaysia

**Keywords:** cranial nerve, malignant otitis externa, nasopharyngeal carcinoma, skull base osteomyelitis

## Abstract

**Objective:**

The primary objective of this study is to review the clinical parameters associated with skull base osteomyelitis (SBO), with a secondary aim of studying their association with patient outcomes 1 and 6 months after treatment initiation.

**Study Design:**

This is a single‐center restrospective observational study.

**Setting:**

The study was conducted from January 2018 to December 2022 at the University Malaya Medical Center in Kuala Lumpur.

**Methods:**

Patients aged over 15 years with a diagnosis of SBO were included in the study. Clinical parameters, investigations, and follow‐up records were recorded. The disease outcomes were analyzed at 1 and 6 months after treatment initiation using multivariable analyses.

**Results:**

The study identified 31 patients with SBO, the majority of whom were elderly males with comorbidities such as diabetes and hypertension. Otalgia and otorrhea were the most common symptoms, and computed tomography scans were used for diagnosis. *Pseudomonas aeruginosa* was the most commonly identified pathogen, and intravenous broad‐spectrum antimicrobials were used to treat all patients. Surgical intervention was required for 25% of patients, and underlying ischemic heart disease, anemia, and single nerve palsy were significantly associated with an unfavorable prognosis. Patients with higher body mass index and elevated C‐reactive protein showed poorer outcomes after 1 and 6 months of treatment, respectively.

**Conclusion:**

Early recognition, prompt treatment, better control of comorbidities, nutrition, and monitoring can improve SBO outcomes and reduce complications. Therefore, as the prevalence of SBO increases, diagnostic criteria or management guidelines should be established to guide the best clinical practice.

Skull base osteomyelitis (SBO) is a rare and potentially life‐threatening condition with high morbidity and mortality. It is often associated with inadequate treatment of otogenic infection, especially in diabetic patients. It then extends and causes inflammation of the bones of the base of the skull. SBO can be divided into typical (lateral) SBO and atypical (central) SBO.^1^, ^2^ Typical SBO is located in the temporal bone and arises from otogenic infection, such as otitis externa. *Pseudomonas aeruginosa* the most common causative organism, which primarily occurs in elderly patients with underlying diabetes mellitus. Atypical SBO is less common and involves the sphenoid and occipital bones without preceding otogenic infection.^2^, ^3^

The clinical manifestations of SBO are generally otalgia, otorrhea, headache and, in advanced cases, conductive hearing loss.^1^, ^4^ The consequences of SBO can be devastating: cranial nerve palsies, meningitis, cerebral venous thrombosis, and intracranial empyema are possible complications.^1^, ^4^, ^5^

Gold standard diagnostic tools for SBO include culture and tissue biopsy. SBO can be treated medically with antibiotics or antifungals based on the causative organism and surgically managed with debridement procedures, such as mastoidectomy. Proximity to the brain, complex craniofacial anatomy, and cosmetic concerns make SBO a therapeutic challenge.^5^, ^6^

Factors contributing to SBO associated with malignant otitis externa include a late diagnosis, delayed or inadequate treatment, failure to respond to antibiotics, and infection in immunocompromised individuals.^7^ Inadequate knowledge and insufficient guidelines raise concerns about diagnostic and therapeutic effects and the course of the disease, which might lead to an unfavorable outcome. This clinical study intends to identify the disease's clinical manifestations, factors affecting the outcomes, and complications. These may assist clinicians in diagnosing SBO early and providing prompt treatment to patients for better prognosis and higher survival rates.

## Methodology

### Study Population

This single‐center observational retrospective study was conducted over a period of 5 years, from January 2018 to December 2022, at the University Malaya Medical Center in Kuala Lumpur. The study was approved by the University Malaya Medical Center Research Ethics Committee (202376‐12646). Patients aged over 15 years with a diagnosis of SBO were included in the study, and a total of 36 patients were identified. However, 5 patients were excluded due to inadequate data and a change in diagnosis. The diagnosis of SBO was based on a combination of clinical features, biochemical blood tests, tissue biopsy, tissue or swab culture, and imaging, including contrasted computed tomography (CT) and magnetic resonance imaging (MRI). The clinical diagnosis criteria resemble those of malignant otitis externa set forth by Cohen and Friedman, where the clinical features of pain, edema, presence of exudate, and positive findings from imaging are combined with failure to improve after 2 weeks of antibiotics.^8^ Follow‐up assessments were performed using the same methods.

### Parameters Assessed

We collected clinical data from electronic medical records of hospitalized patients, which included demographic information, comorbidities, clinical presentation, physical examination results, laboratory and radiological findings, as well as management interventions. Additionally, we analyzed hospitalization, complication, and mortality rates.

**Figure 1:**
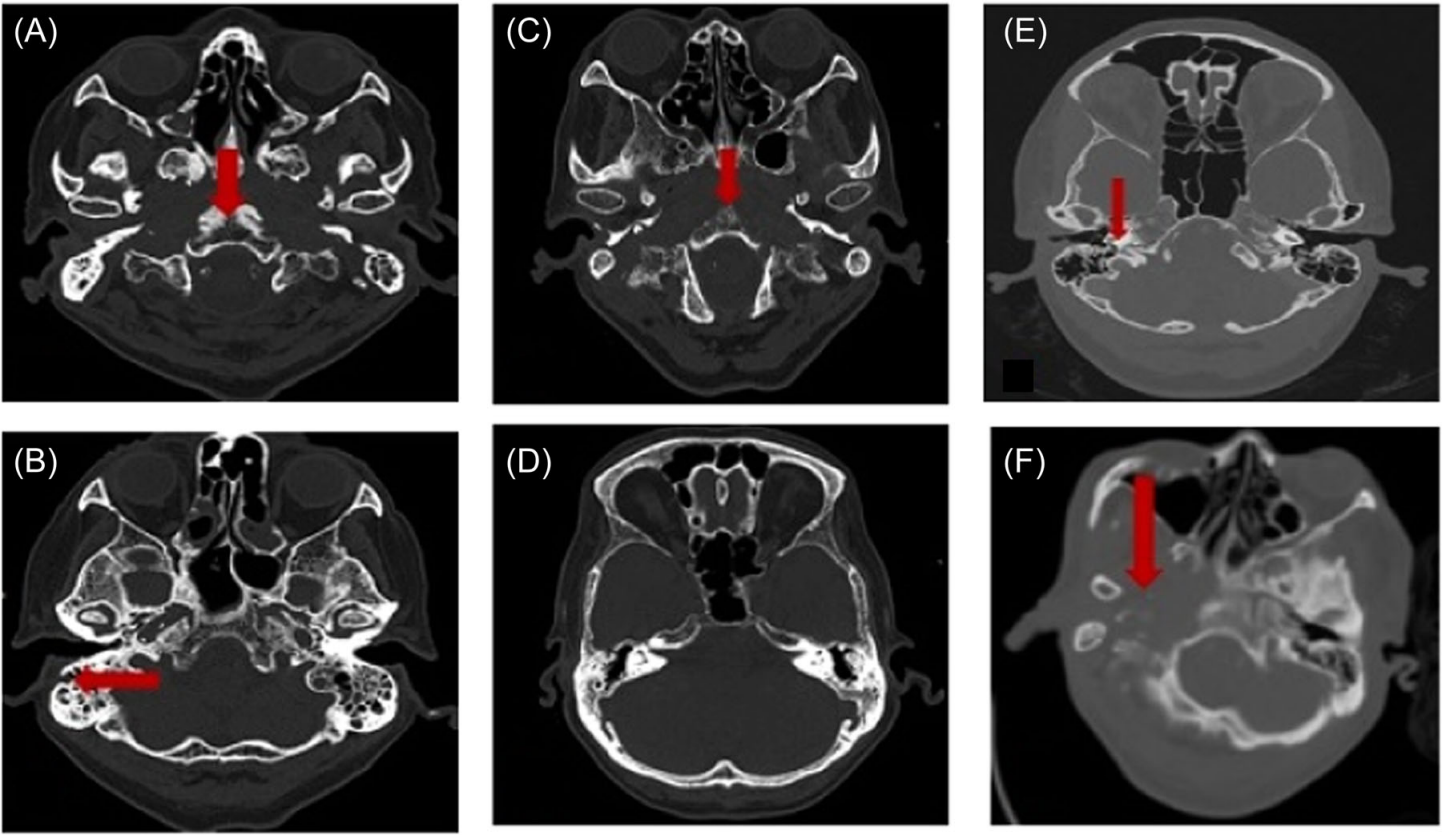
Figures (A–F) show multiple axial cuts of a computed tomography scan imaging of skull base osteomyelitis. Red arrow indicates the site of erosion.

In terms of the site of SBO, we categorized the location anatomically. Central SBO affects the sphenoidal and occipital bones, while lateral SBO pertains solely to any part of the temporal bone. A combination of both central and lateral SBO is known as mix SBO, which is illustrated in Figure 1.

### Disease Outcome

We classified our disease outcomes as “improved” versus “not improved.” The “not improved” outcomes are of concern: they include conditions without clinical or laboratory improvement, disease progression, or death. The improved outcome is complete resolution or improvement in clinical symptoms, laboratory parameters, and imaging findings. The 2 groups were compared in relation to the clinical characteristics of the subjects recruited. The disease outcomes were analyzed at 1 month and 6 months after treatment initiation.

### Statistical Analysis

Statistical analysis was conducted using the Statistical Package for the Social Sciences software, version 27.0 (IBM Corp). The age (years) of subjects, body mass index (BMI), comorbidities score, blood parameters, duration of prediagnostic symptoms, hospital stay, readmission rate, and treatment duration were noted as continuous variables. The presence of any comorbid condition, gender, culture growth, symptomatology, imaging findings, treatment types, and complications were considered as categorical variables.

Continuous variables were demonstrated as mean ± standard deviation. Student's *t* test for continuous variables and Pearson *χ* ^2^ test for categorical variables were used to chart the comparisons of these variables between those who improved and not improved. *P* value of less than .05 is considered statistically significant.

## Results

A total of 31 patients were diagnosed with SBO. The majority (24; 77.4%) were elderly males, with a mean age of 66.06 ± 10.063 years (38‐81) and a mean BMI of 23.9 ± 4.5 kg/m^2^. SBO was predominantly diagnosed in Indian populations (51.6%), followed by Malay (29.0%), Chinese (16.1%), and others (3.2%). All patients had diabetes mellitus as a comorbid condition, with a mean glycated haemoglobin (HbA1c) of 8.37 ± 2.01 mmol/mol. Additionally, 80.6% of patients had hypertension, 74.2% had anemia, and 48.4% had ischemic heart disease (IHD).

Patients diagnosed with SBO commonly exhibit a combination of symptoms, predominantly including otalgia (93.5%) and otorhea(64.5%). All patients experienced ear‐related symptoms except for one who only had a headache. This patient, however, had both central and lateral SBO. Only 2 patients had purely central disease, and both had multiple cranial nerve palsies. Among the patients with single nerve involvement, all had facial nerve palsy. Four patients had multiple cranial nerve palsies with interestingly varied nerve involvement. The average duration of symptoms prior to diagnosis was 35 ± 28 days. Physical examination commonly reveals the presence of granulation (51.6%), a swollen canal (41.9%), and a perforated tympanic membrane (32.3%).

Radiological investigations play a crucial role in diagnosing SBO by accurately identifying and determining the extent of the condition. All patients underwent contrast CT scans, while MRI was reserved for those suspected of intracranial complications. CT imaging can readily detect fluid opacification and bone erosion or disintegration, which are typical radiological manifestations of SBO. It is worth noting that a large percentage of detected SBO cases were lateral SBOs (73.3%), and a significant number of cases involved only a single bone (64.5%).

Out of 31 patients, 22 (70.97%) had positive cultures. Out of these, 17 (54.84%) were bacterial, 3 (9.68%) were fungal, and 2 (6.45%) were a mixture of bacterial and fungal or bacterial and viral infections. *P. aeruginosa* was the most commonly identified pathogen and was found in 10 patients (35.48%). *Staphylococcus* species were detected in 6 patients, including methicillin‐resistant *Staphylococcus aureus*, methicillin‐susceptible *Staphylococcus aureus*, and *Staphylococcus capitis*. Additionally, *Candida* species were identified in 4 patients, *Aspergillus flavus* in 2 patients, and *Klebsiella pneumonia* in 2 patients.

All patients were treated both intravenous follow by oral broad‐spectrum antimicrobials. Ciprofloxacin was prescribed to the majority of patients (67.7%), while 13 patients received ceftazidime. Of the patients, 25% underwent surgical intervention, with 4 procedures involving the removal of bony tissue, such as mastoidectomy or modified radical mastoidectomy, and the other 4 involving soft tissue abscess drainage and debridement. The average duration of antimicrobial therapy was 36.16 ± 17.52 days, with a mean of 20 ± 19.09 and 16.06 ± 14.18 days of intravenous and oral antibiotics, respectively. The mean hospital stay was 20.74 ± 19.15 days. Following discharge, 45.2% of patients required readmission within 2 weeks.

The overall results are listed in Table 1.

**Table 1:** Demographic, Clinical, and Radiological Data of Patients With Skull Base Osteomyelitis

	N = 31
Comorbid conditions	
Diabetes	31 (100%)
Hypertension	25 (80.6%)
Ischemic heart disease	15 (48.4%)
Anemia	23 (74.2%)
Charlson score comorbidities > 2	28 (90.3%)
Body mass index > 25.0, kg/m^2^	10 (32.25%)
Laboratory parameters	
Total leukocyte count/mm^3^ (4000‐11,000) (mean ± SD)	11.47 ± 4.57
CRP, mg/L	27.33 ± 35.98
HbA1c, %	8.37 ± 2.01
ESR, mm/h	52.20 ± 21.09
Albumin, g/L	28.71 ± 6.57
Sign and symptoms	
Otalgia	29 (93.5%)
Otorrhea	20 (64.5%)
Swollen canal	13 (41.9%)
Tympanic membrane perforation	10 (32.3%)
Granulation	16 (51.6%)
Cranial nerve palsy	
Single nerve palsy	6 (19.4%)
Multiple nerves palsy	4 (12.9%)
Radiological findings	
Bone involvement	
Single bone	20 (64.5%)
Multiple bones	11 (35.5%)
Types of SBO	
Central	2 (6.7%)
Lateral	22 (73.3%)
Mixed	7 (22.6%)
Management	
Surgery	8 (25%)
Bony	4 (12.5%)
Soft tissue	4 (12.5%)
Duration of antimicrobial usage (mean days ± SD)	36.16 ± 17.52

Abbreviations: CRP, C‐reactive protein; SBO, skull base osteomyelitis; ESR,erythrocyte sedimentation rate; HbA1c, glycated haemoglobin.

Two patients were excluded from the analysis of disease outcome due to loss to follow‐up at 6 months. The results of the study showed that patients with underlying IHD had a significantly worse prognosis (*P* = .045) compared to those without, as indicated in Table 2. The categorical variables at 6 months revealed that underlying IHD (*P* = .016), anemia (*P* = .035), and single nerve palsy (*P* = .017) were also significantly associated with an unfavorable prognosis at 6 months, as shown in Table 3.

**Table 2:** Relationship Between Categorical Variables and Disease Outcome at 30 Days

	Outcome in 30 days, n (%)			
Categorical variables	Improved (n* *= 14)	Not improved (n* *= 17)	Pearson *χ* ^2^	*P* value
Gender				
Male	9 (64.3)	15 (88.2)	2.519	.198
Female	5 (35.7)	2 (11.8)		
Comorbids				
Diabetes on oral hypoglycaemic agents	12 (85.7)	11 (64.7)	1.770	.240
Diabetes on insulin	5 (35.7)	9 (52.9)	0.920	.337
Hypertension	9 (64.3)	16 (94.1)	4.377	.067
Stroke	3 (21.4)	4 (23.5)	0.190	1.000
Ischemic heart disease	4 (28.6)	11 (64.7)	4.014	.045
Anemia	14 (100.0)	9 (52.9)	1.309	.412
Culture growth	11 (78.6)	11 (64.7)	0.716	.456
Complications				
Single nerve palsy	1 (7.1)	5 (3.3)	2.439	.185
Multiple nerve palsies	1 (7.1)	3 (17.6)	0.754	.607
Imaging findings				
Single bone involvement	5 (35.7)	9 (52.9)	0.920	.337
Multiple bone involvement	4 (28.6)	6 (35.3)	0.159	1.000
Lateral skull involvement	12 (85.7)	11 (64.7)	0.368	.689
Intervention				
Surgery	11 (78.6)	11 (64.7)	0.716	.456
Types of surgery			3.789	.150
Soft tissue	0	4 (23.5)		
Bony	2 (14.3)	2 (11.8)		
ICU stays	1 (7.1)	2 (11.8)	0.188	1.000

Abbreviation: ICU, intensive care unit.

**Table 3:** Analysis of Categorical Variables in Relation to Disease Outcomes at 6 Months

	Outcome at 6 months, n (%)			
Categorical variables	Improved (n = 15)	Not improved (n = 14)	Pearson *χ* ^2^	*P* value
Gender				
Male	10 (66.7)	13 (92.9)	3.027	.169
Female	5 (33.3)	1 (7.1)		
Comorbids				
Diabetes on oral hypoglycaemic agents	12 (80.0)	9 (64.3)	0.895	.427
Diabetes on insulin	6 (40.0)	8 (57.1)	0.852	.356
Hypertension	11 (73.3)	13 (92.9)	1.934	.330
Stroke	4 (26.7)	3 (3.4)	0.109	1.000
Ischemic heart disease	4 (26.7)	10 (71.4)	5.811	.016
Anemia	8 (53.3)	13 (92.9)	5.663	.035
Culture growth	12 (80.0)	9 (64.3)	0.895	.427
Complications				
Single nerve palsy	0	5 (35.7)	6.473	.017
Multiple nerve palsies	2 (13.3)	2 (14.3)	0.006	1.000
Imaging findings				
Single bone involvement	6 (40.0)	7 (50.0)	0.293	.588
Multiple bone involvement	5 (33.3)	5 (35.7)	0.018	1.000
Lateral skull involvement	12 (80.0)	8 (57.1)	1.163	.410
Management				
Surgery	13 (86.67)	9 (64.3)	1.981	.215
Types of surgery			2.029	.363
Soft tissue	1(1.6)	2(14.3)		
Bony	1(2.1)	3(21.4)		
ICU stays	1 (6.7)	2 (14.3)	0.453	.598

Abbreviation: ICU, intensive care unit.

Upon further analysis of the continuous data presented in Table 4, it was found that patients with higher BMI (*P* = .048) and elevated levels of C‐reactive protein (CRP), with an average of 44.95 ± 47.02 mg/L (*P* = .021), displayed poorer outcomes at 1 month and 6 months after the initiation of treatment, respectively. It is essential to note that an elevated CRP value is defined as one that exceeds 5.00 mg/L. The overall results revealed that 13 patients experienced disease resolution at 1 month. Out of 16 patients who had disease progression, unfortunately, 3 patients succumbed to the disease at 6 months despite aggressive antibiotic treatment and surgical intervention.

**Table 4:** Comparison of Continuous Variables With Disease Outcomes at 1 and 6 Months

	Outcome in 1 month (mean ± SD)			Outcome in 6 months (mean ± SD)		
Continuous variables	Improved	Not improved	*P* value	Improved	Not improved	*P* value
Age	63.21 ± 12.18	68.41 ± 7.51	.178	63.13 ± 11.72	69.14 ± 8.06	.122
BMI, kg/m^2^	22.23 ± 3.79	25.26 ± 4.65	.048	22.24 ± 3.73	24.70 ± 4.25	.142
Charlson score comorbidities	4.07 ± 1.86	5.06 ± 2.19	.200	4.40 ± 1.99	5.00 ± 2.29	.457
Blood parameters						
Total white cell counts, mm^3^	11.64 ± 5.11	11.33 ± 4.23	.853	11.77 ± 5.26	11.84 ± 3.80	.813
Raised CRP, mg/L	15.91 ± 14.35	36.72 ± 45.29	.653	11.64 ± 11.47	44.95 ± 47.02	.021
HbA1c, %	8.69 ± 2.17	8.10 ± 1.88	.422	8.31 ± 2.16	8.57 ± 2.00	.555
ESR, mm/h	48.36 ± 22.34	55.56 ± 20.03	.360	46.47 ± 18.64	58.15 ± 19.41	.117
Albumin, g/L	29.71 ± 7.04	27.88 ± 6.24	.449	30.40 ± 7.23	27.14 ± 6.024	.200
Duration of prediagnosis, d	34.07 ± 18.86	30.50 ± 19.90	.637	30.60 ± 17.81	45.36 ± 35.82	.198
Duration of antibiotics, d	34.79 ± 19.28	37.69 ± 16.44	.699	35.27 ± 20.00	38.64 ± 14.52	.609
Hospital admission						
Length of hospital stay, d	18.93 ± 19.37	22.24 ± 19.44	.710	20.40 ± 19.98	22.50 ± 19.95	.878
Days before readmission, d	18.43 ± 31.94	21.24 ± 44.60	.739	8.87 ± 17.521	34.71 ± 52.011	.100
Number of readmissions	0.64 ± 1.15	1.35 ± 1.90	.316	0.60 ± 1.30	1.64 ± 1.87	.052

Abbreviations: BMI, body mass index; CRP, C‐reactive protein; ESR, erythrocyte sedimentation rate; HbA1c, glycated haemoglobin.

## Discussion

SBO is a complex, serious condition that increases the risk of significant morbidity and mortality. Unfortunately, there are currently no established guidelines for managing SBO. Therefore, treatment remains challenging due to the limited opportunities for surgical intervention with often unknown pathogens and unknown optimal duration of antimicrobial therapy.^9^, ^10^, ^11^

Notably, SBO primarily affects males, with a male‐to‐female ratio of 3.4:1. There was a marked predominance of SBO among the elderly in their sixth decade, with a mean age of 66.06 ± 10.063 years, which is in concordance with other reports.^10^, ^11^ In our study, a higher BMI was significantly associated with a poorer prognosis at 1 month of treatment commencement (*P* = .048). Some authors suggest that this may be due to obesity‐induced vascular insufficiency, which can reduce oxygen tension and collagen production, leading to a lower capacity to fight infection and inadequate support for the healing process.^12^, ^13^, ^14^

Poorly controlled diabetes mellitus was a significant risk factor for SBO. Leukocyte function is impaired in diabetic patients and therefore, they are more susceptible to infection and significant wound inflammation.^15^ Hypertension was common in patients with SBO (80.6%). Chronic hypertension affects cerebral circulation due to effect of Angiotensin II as the primary cause of oxidative stress, endothelial dysfunction, and increased tone in cerebral arteries.^16^ Poor circulation eventually worsens osteomyelitis and delays wound healing.^17^ On further analysis, IHD was also one of the critical risk factors for SBO significantly associated with poorer prognosis at 1 month (*P* = .045) and 6 months (*P* = .016). Patients with underlying IHD generally have narrow or blocked vessels, which contributes to poor circulation and perfusion with nutrients and oxygen to the area of infection.^18^

Some previous reports have indicated that the most common presentation of SBO is headache, followed by otalgia and otorrhea.^6^, ^9^, ^11^ However, our study found that otalgia was the predominant symptom (93.5%), followed by otorrhea (64.5%) and headache (29%). Our findings also suggest that a single nerve palsy may result in poorer outcomes at 6 months (*P* = .017), potentially due to deep infection along the skull base.^19^

At our institution, CT scan is the preferred imaging technique for diagnosing and monitoring SBO. CT scans can detect small changes in bone density and identify the extension of the infection into soft tissue.^20^ The type of SBO is determined based on CT scan findings. Goh et al suggest that MRI with contrast is helpful, especially in differentiating advanced nasopharyngeal cancer and SBO, where the most prevalent findings in SBO were a lateral extension, increased T2 signal in adjacent soft tissues, lack of architectural distortion and enhancement greater than or equal to mucosa.^21^

In addition to radiological imaging, obtaining cultures is a recommended practice to determine the causative pathogen and provide appropriate antimicrobial treatment. Our findings showed *P. aeruginosa* was the most frequently identified pathogen.

Blood parameters also played a role in predicting the outcomes of SBO patients. Patients with anemia on diagnosis generally had a poorer outcome at 6 months (*P* = .035). Anemia causes a reduction in the oxygen‐carrying capacity of the blood.^22^ Oxygen is required for cell proliferation, bacterial defence, angiogenesis, epithelialization, collagen synthesis, and granulation tissue formation. The healing process requires a vast amount of oxygen to maintain the production of adenosine triphosphates (ATPs) and thus sustain a high energy level to support cellular recovery.^23^, ^24^ The research findings suggest that patients who were admitted with high levels of CRP are at a higher risk of an unfavorable outcome within 6 months following treatment initiation (44.95 ± 47.02 mg/L) (*P* = .021). The study also found that the standard deviation of elevated CRP was higher than the mean, which indicates a wide distribution of CRP values ranging from 5.1 to 131.3 mg/L. As CRP serves as an inflammatory marker, it aids in determining the extent of the infection and the efficacy of the treatment administered. While investigations such as white cell count, erythrocyte sedimentation rate, albumin, and HbA1c did not exhibit any correlation with the disease outcomes, they provide valuable insights into the overall health status of the patient. Therefore, optimizing these parameters could prove beneficial in facilitating the patient's recovery.

The management of SBO generally involves aggressive antimicrobial therapy with antibiotics or antifungals, with or without surgical debridement. Broad‐spectrum antibiotics such as ciprofloxacin or ceftazidime are commonly used. Nevertheless, the initial selection of antibiotics can vary contingent upon the attending physician's predilection and experience, while awaiting the culture and sensitivity results. The average duration of antimicrobial therapy was 36.16 ± 17.52 days, with a minimum duration of 6 weeks, which is consistent with similar reports.^6^, ^25^ However, impaired microvascular circulation limits white blood cell transportation and restricts the delivery of adequate antibiotics and nutrients to the infected area.^26^, ^27^ Surgical intervention was performed if the disease was severe and had an inadequate response to antimicrobial therapy.^7^, ^9^, ^10^, ^28^ Our study does not define a biopsy alone as surgery. Subjects with a poor response to medical antibiotics were referred for surgery, either soft tissue (eg, soft tissue debridement or collection drainage) or bony surgery (eg, modified radical mastoidectomy or cortical mastoidectomy). However, surgery was not a significant parameter associated with patient's outcome in this paper.

There are a few noteworthy limitations to consider. First, this study is based on a small sample of patients from a single institution. Second, given that our institution is a tertiary referral center, the majority of cases are more intricate and advanced. Finally, further data analysis on antimicrobial and antifungal treatments will impact the study results. However, the wide variability in medication usage poses challenges for our data analysis. Therefore, it is important to keep in mind that the results may not fully reflect the true prevalence of the disease within society.

## Conclusion

SBO presents diagnostic and therapeutic challenges as there are no clinical criteria to diagnose and manage this disease. Our study indicates that underlying complications, the presence of complications upon presentation, poor nutrition, IHD, and obesity are linked to worse disease outcomes. It is imperative to conduct further analysis with a larger number of patients to enhance disease outcomes and reduce the risk of complications. Compliance with disease monitoring is essential for promoting recovery and preventing recurrence.

## Disclosures

### Competing interests

None.

### Funding source

None.

## Data Availability

Electronic medical record of the hospital.
